# Improving the Sensitivity of Protein Quantification by Immunoaffinity Liquid Chromatography—Triple Quadrupole Mass Spectrometry Using an Iterative Transition Summing Technique

**DOI:** 10.1021/acs.analchem.3c04598

**Published:** 2024-08-26

**Authors:** Jay S. Johnson, Joe Palandra, Nikolaos Psychogios, Jason M. Walsh, Hendrik Neubert

**Affiliations:** Pharmacokinetics, Dynamics & Metabolism (PDM), Pfizer, Andover, Massachusetts 01810, United States

## Abstract

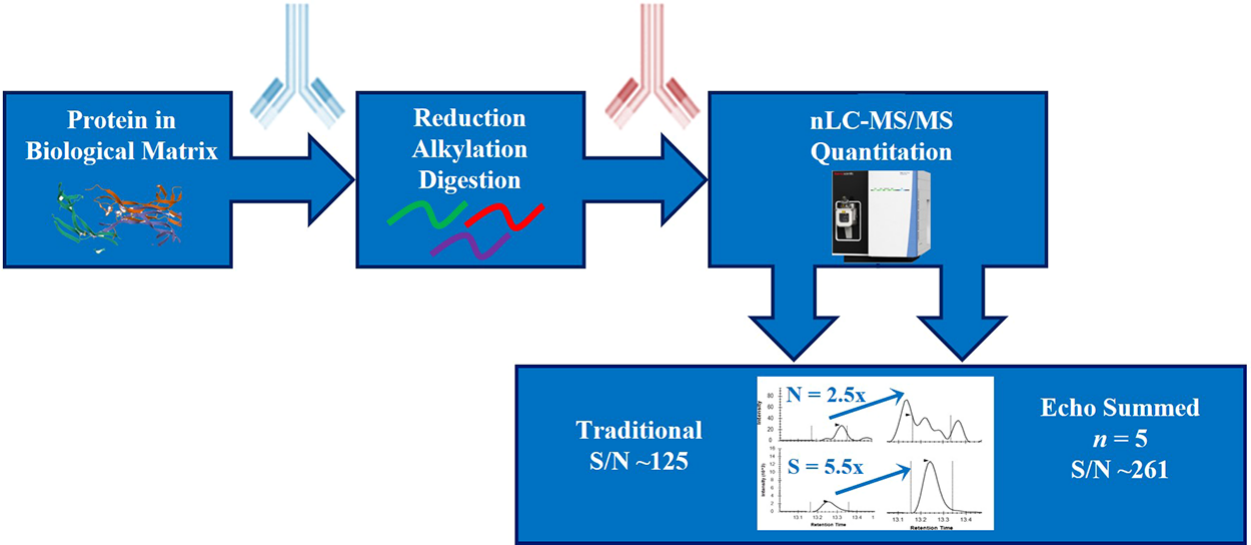

The desire to reach ever-diminishing lower limits of quantification (LLOQ) to probe changes in low abundance protein targets has led to enormous progress in sample preparation and liquid chromatography–tandem mass spectrometry (LC-MS/MS) instrumentation. To maximize signal and reduce noise, many approaches have been employed, including specific immunoaffinity (IA) enrichment and reducing the LC flow to the nanoflow (nLC) level; however, additional sensitivity gains may still be required. Recently, a technique termed “echo summing” has been described for small-molecular-weight analytes on a triple quadrupole (QqQ) MS where multiple iterations of the same, single selected reaction monitoring (SRM) transition are collected, summed, and integrated, yielding significant analyte dependent signal-to-noise (S/N) improvements. Herein, the direct applicability of echo summing to protein quantification by sequential IA combined with nLC-MS/MS (IA-nLC-MS/MS) is described for a beta nerve growth factor (NGF) and a soluble asialoglycoprotein receptor (sASGPR) assay from human serum. Five iterations of echo summing outperformed traditional collection in relative average accuracy (−1.5 ± 7.7 vs −41.7 ± 10.7% bias) and precision (7.8 vs 18.4% coefficient of variation (CV)) of the low-end quality control (QC) sample (*N* = 4) for NGF and improved functional sensitivity of serially diluted serum QC samples (*N* = 5 each population) approximately 2-fold (1.96 and 2.00-fold) for two peptides of sASGPR. Echo summing also extended the minimum quantifiable QC level for sASGPR 4-fold lower. Similar gains are believed to be achievable for most protein IA-nLC-MS/MS assays.

Quality protein quantification assays are in high demand across a range of fields including biomarker discovery,^1,2^ pharmacokinetic/pharmacodynamic (PK/PD) analysis of biotherapeutic and target,^3−5^ and diagnostics.^6,7^ The proteins targeted in these assays can have low concentrations resulting from biomarker degradation/inhibition, biotherapeutic/target clearance, disease state biology, and other factors. Gaps in the ability to accurately quantify changes in concentration can impact the scientific conclusions that are drawn. In instances where biotherapeutic dose is low and clearance is fast, for example, PK concentrations can rapidly drop below the lower limit of quantification (LLOQ), leading to truncation of the PK profile.^4,5^ Accordingly, the field of protein quantification is continually pushing for more sensitive and selective methods to fill these gaps.

Liquid chromatography (LC) combined with triple quadrupole liquid chromatography/mass spectroscopy (QqQ) is routinely used for targeted protein quantification after tryptic digestion,^3,4,6−14^ due to the high specificity resulting from the temporal separation of the resulting peptides by LC, mass isolation in the first quadrupole (precursor), and mass selection in the third quadrupole of the discrete fragments of interest (product) post-collision in the second quadrupole. The specificity and sensitivity can be increased drastically by employing an IA purification step to deplete the deep background complexity in biological matrices and enrich and concentrate the target. The affinity purification step can be accomplished in a multiplexed fashion at the protein,^3,4^ peptide,^6−9,29^ or by combining both sequentially^10−14^ prior to LC-MS/MS. These so-called “IA-LC-MS/MS” assays offer attractive figures of merit and LLOQs down to the tens of pg/mL range.

In pursuit of more sensitivity, recent IA-LC-MS/MS assays have reduced the eluate flow to nLC levels of <1 μL/min,^9−14^ achieving an approximate 10-fold improvement in LLOQ; however, situations arise where proteins of interest are still not detectable.^10^ Another underutilized technique that can have a profound effect on sensitivity in QqQ mass spectrometry centers on how discrete transitions are collected and chosen to be summed and integrated for quantification.

In small-molecule workflows, a single precursor to product transition is typically monitored and used for quantification due to the predominant formation of only a single stable product ion. Unlike small molecules, peptides form multiple stable and strong product ions due to their size and characteristic fragmentation patterns. Conventionally, a single peptide transition offering the needed signal with minimal noise is chosen for quantification while additional sensitive product ions are collected to serve as confirmatory, qualitative transitions.^3,8,10,12−14^ However, multiple discrete, product ions from one or more precursor or related precursors can also be monitored and their signals summed, when beneficial, for quantification.^4,7^

Recent publications have reported another technique for transition collection in small-molecule^16−21^ and intact protein^15^ assays where multiple iterations of the same, single transition are collected, summed, and integrated. This technique, colloquially termed “echo summing”, has been documented to yield significant analyte-dependent signal-to-noise ratio ( S/N) improvements for small molecules ranging from 2- to 4-fold, utilizing various sample preparation techniques. In echo summing, the signal presented in the summed chromatogram will increase linearly by a factor of the number of iterative transitions collected (*n*) and S/N will increase by *n*/√*n*. The improvement in S/N is a consequence of the signal being multiplied and low level, nonreproducible noise being averaged out over the multiple summing iterations. The nature of summing the same transition multiple times implicitly ensures that the signal gain will outpace that of the noise, leading to S/N improvement. Pauwels et al. confirmed in their work with the small-molecule everolimus that, in instances where only a single transition is echo-summed, the increase in the (S/N) is proportional to *n*/√*n* or √*n*.^17^ Subsequent studies by Movassaghi et al. found no conclusive evidence of S/N increasing by the √*n* proportion, despite improved sensitivity and an up to 3-fold reduction in LLOQ for the halogenated compound 1DCV.^21^

Collecting additional transitions for improving sensitivity, the central concept of echo summing must outpace the competing loss in sensitivity resulting from the faster sampling frequency. As the number of individual transitions collected increases in a fixed cycle time, the time spent collecting each transition, known as the dwell time, decreases proportionally to the increase in the total number of transitions. Accordingly, dwell time theory suggests the additional transitions collected (*n*) in echo summing should decrease the S/N value by a factor of 1/√d*t*, erasing the sensitivity gains predicted by the √*n* relationship.^22,23^ This concept is demonstrated graphically in Figure S1. A statistical model of echo summing, presented by Lytle et al., has arrived at a similar conclusion that no tangible benefits should be achieved using echo summing.^24^ Considering dwell time theory limitations, the disparate conclusions from these studies with small molecules, and the potential for increased sensitivity, we found it prudent to conduct our own experiments with echo summing and its applicability to sequential IA-nLC-MS/MS for the quantification of NGF and sASGPR proteins in human serum.

## Experimental Section

Previously published methodology and validation of the model NGF assay^12−14^ utilizing sequential protein and peptide IA was followed in this experiment, unless explicitly noted. A similar methodological approach was utilized to assess the functional sensitivity of sASGPR using a nonvalidated assay. Further information on the calibration curve, quality control (QC) sample construction, and additional LC and MS operating parameters is documented in the Supporting Information.

The transition details utilized for the traditional collection and echo summing methodology are documented in Tables S1 and S2. For NGF, traditional transition collection consists of six transitions in a 0.65 s cycle time. The echo summing collections consists of five iterations (*n* = 5) of the light and SIL trace of the quantitative y7 transition and a single iteration of the qualitative transition pair in the same cycle time. For sASGPR, traditional transition collection consists of three transitions for each peptide in a 0.65 s cycle time. The echo summing consists of five iterations (*n* = 5) of the light and SIL trace of the quantitative y6 transition for SLESQLEK and the quantitative y2 transition for QFVSDLR and a single iteration of the qualitative transition pair collected in the same cycle time. The additional echo summing transitions result in a decrease in d*t* of ∼2.4 (107 vs 45 ms, 53 vs 22 ms) for both assays, presenting a situation that is not covered by previously published echo summing models but common in peptide analysis whereby *n* ≠ d*t*.

To enable the QqQ MS to collect the echo summing transition multiple times, the mass of the product ion is varied by a small increment (0.004 Da) centered around the actual mass. The variation of the mass must be large enough for the instrument to collect the data as a discrete transition but small enough to collect the entire Gaussian mass envelope and should be optimized for the specific MS model utilized. We also echo-summed the SIL transition in the same manner as the light trace to allow statistical assessment of ion ratio patterns and to prevent the introduction of any bias in the signal area ratios.

Each sample well was injected and run by traditional collection first, followed immediately by a second injection of the same sample using echo summing (NGF) as part of a single batch. For sASGPR, the collection order was purposefully flipped (first, echo summing; second, traditional collection) to remove run order as a potential confounding variable in the results. Calibration curves are assayed at the start and end of the sample set to bracket the QC samples. QC samples are assayed in ascending order of the predicted concentration. Matrix blanks are interspersed throughout the experiment and assayed when the signal is presumed to be blank. Accordingly, each sample was assayed by both collection methods in a temporal succession.

Data for all samples utilizing a collection type was imported into Skyline 23.1.0.380 (University of Washington, Seattle, WA) using the molecule interface for interpolated transformation, summation, integration, and determination of signal area ratios between the light and SIL peptide. The transition list was entered manually in Skyline so that it reflected the transition details and masses for each collection technique. Importation of echo summing data should result in discrete chromatograms for all masses collected, and each chromatogram is checked for this attribute prior to summing. Signal area ratios were exported to Excel and standard curves generated by plotting the signal area ratio against concentration of the calibrants and fitted with a weighted quadratic line of best fit. The weighting utilized was chosen using the least-squares method. Concentrations reported throughout are derived from back-calculation using the calibration curves. The accuracy of the QC samples was calculated by comparison to the nominal concentration expected based on the serum dilution or spike addition and calculated as a relative accuracy bias throughout. The nominal endogenous concentration is calculated by averaging the back calculated concentrations for all replicates of the endogenous serum QC (QCE), irrespective of collection method (*N* = 8 replicates for NGF, *N* = 10 replicates for sASGPR). Assay accuracy was assessed by calculating the average bias and corresponding standard deviation (SD) for each QC population. Accuracy acceptance criteria were set at ±20% and SD less than 20%. Assay precision was assessed by calculating the imprecision as a coefficient of variation (CV) for each QC population. Precision acceptance criteria were set at ±20%, CV less than 20%, and LLOQ at the explicit concentration in which precision reaches the 20% CV threshold. Functional S/N is calculated by arithmetically dividing the maximum height response of the SIL signal in the surrogate matrix blank by the maximum height response in the light trace of the same sample by using the SIL integration boundaries for reference. Any signal present in the light trace is considered to be equivalent to noise. The maximum height response in counts per second for each trace is determined by exporting the chromatographic data under the SIL integration boundaries using Skyline and recording the maximum value present.

## Results and Discussion

Comparisons of calibration curves and statistics for traditional versus echo summing collection of NGF are shown in Figure S2 and Table S3. Selected extracted ion chromatograms for each technique are shown in Figure S3. Echo summing provides precise and accurate quantification of NGF across four replicates of all QC levels due to its enhanced sensitivity (Table 1). For QCE/4, the average accuracy is −1.5 ± 7.7% and precision is 7.8% CV. In comparison, traditional collection results in all four replicates of QCE/4 failing to pass acceptance with an average accuracy of −41.7 ± 10.7% and precision is 18.4% CV. Further failures also occur for three replicates of QCE/2 using traditional collection, but these failures do not push the average accuracy and precision outside of acceptance criteria. Accordingly, acceptable precision and accuracy statistics are preserved with echo summing at a QC concentration 2-fold lower than that of traditional collection (QCE/4 vs QCE/2; 5.20 pg/mL vs 10.39 pg/mL). The loss of low-end quantitative ability using traditional collection is a direct result of the signal not being highly differentiated from the noise, as seen in Figure S3. Echo summing achieves a mean S/N value of 261.4 or 2.08-fold greater than the S/N value of 125.4 achieved for traditional collection. Comparison of the signal for the lowest calibrator (CAL2; 2.5 pg/mL) to the noise present in the matrix blank results in an echo-summing mean S/N gain of 2.09-fold (8.6 vs 4.1). Accordingly, the improvement in S/N as well as the minimum quantifiable QC concentration when echo summing is consistent with the predicted gain of √*n* or 2.24.

**Table 1:**
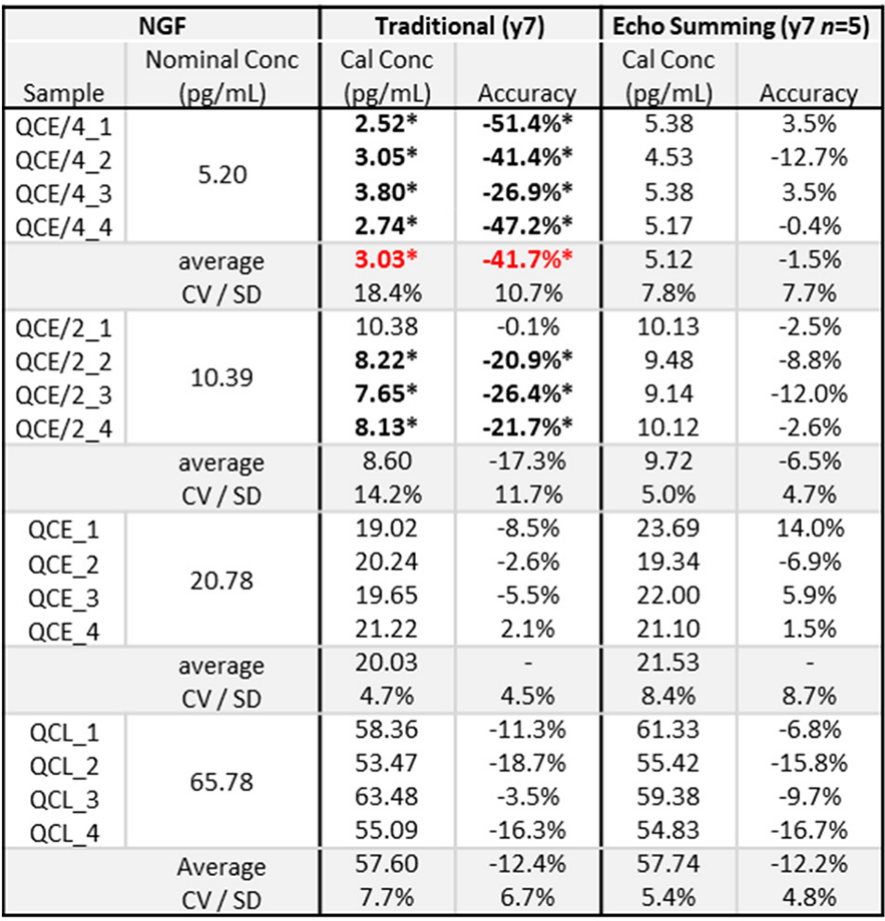
Comparison of QC Accuracy and Precision for Traditional vs Echo Summing of NGF^a^

a Individual values outside of acceptance criteria are bolded and denoted by an asterisk, while average values are shown in a bold red font.

**Figure 1:**
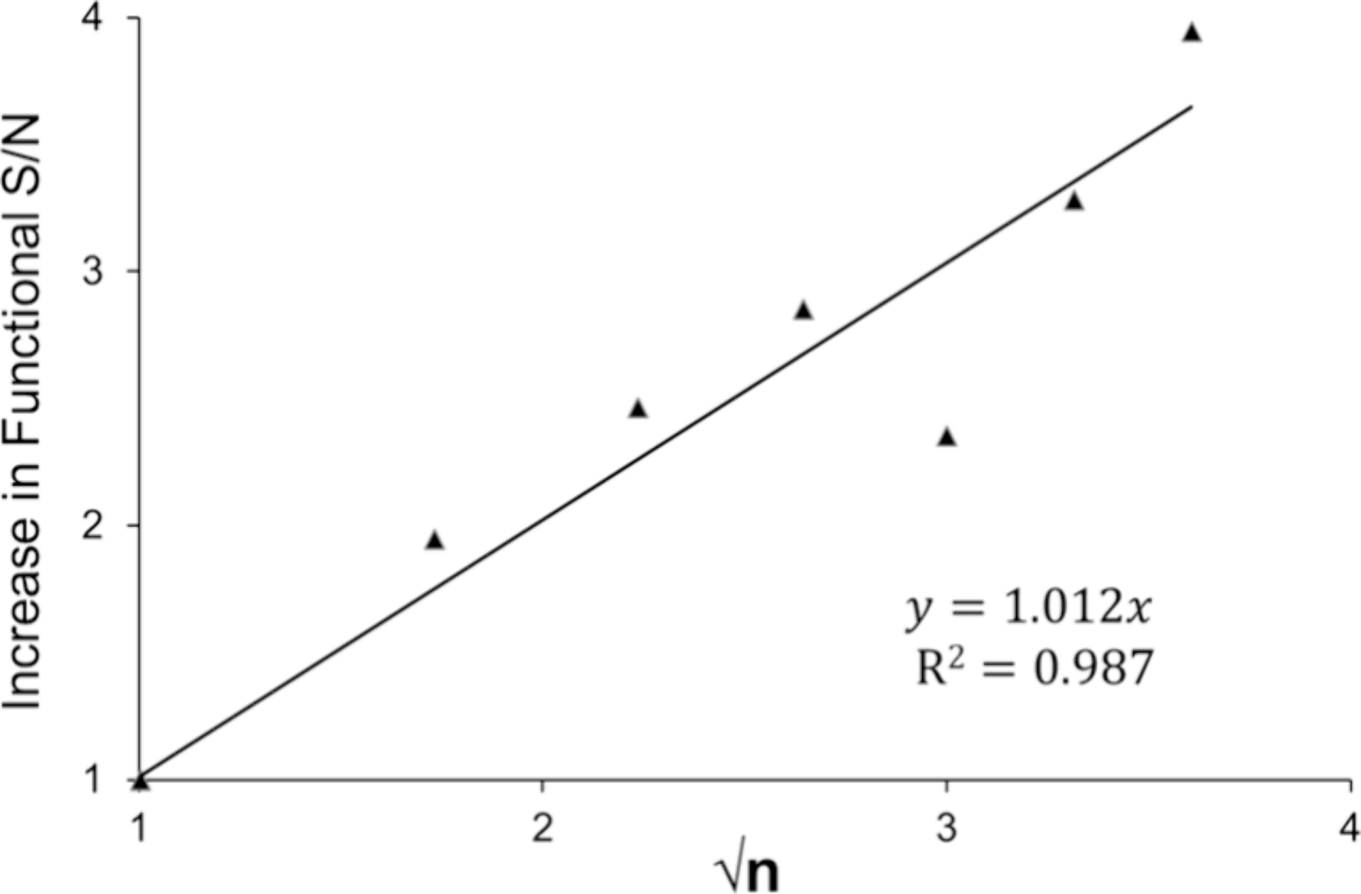
Correlation plot between experimental S/N gains achieved with echo summing and the √*n* model for NGF.

**Figure 2:**
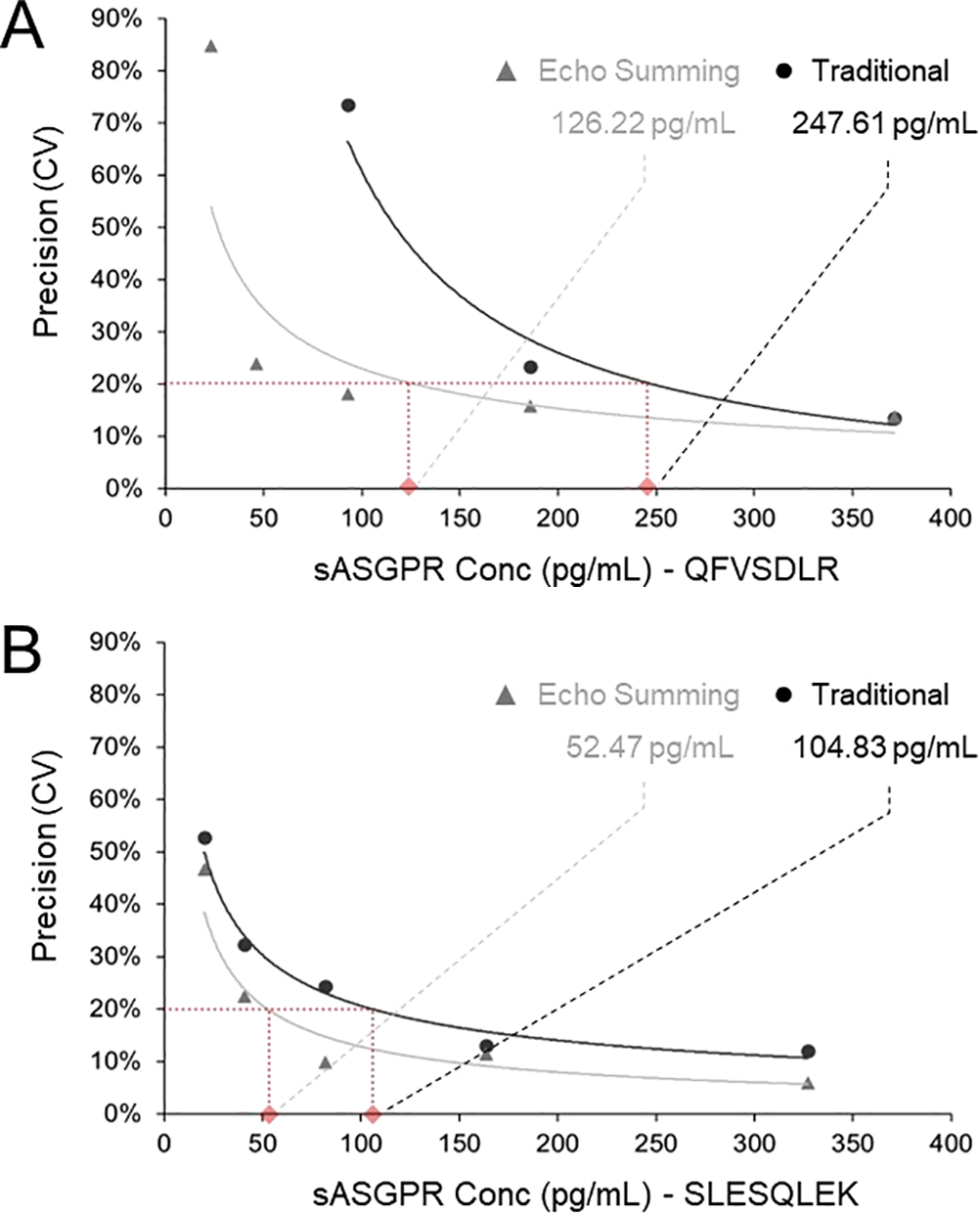
Comparison of precision in the measured sASGPR concentration using (A) the QFVSDLR peptide and (B) the SLESQLEK peptide.

The 5-fold larger peak resulting from echo summing lends itself to more reproducible peak integration at the low end of the quantification range, as reflected in the improved precision statistics. This finding of improved precision, while expected, was not observed in the previous echo summing experiments conducted on small molecules.^17^ The determination of an explicit statistical and LLOQ improvement for echo summing in the proof-of-concept NGF testing cannot be accomplished, as the QC concentration does not drive low enough to pass the established accuracy and precision thresholds of 20% SD and 20% CV (Table 1). However, the NGF testing does provide a framework for the functional sensitivity assessments of sASGPR (see Figure 2 and Table 2, each presented later in this work) as well as an assessment of the functional S/N improvements afforded by echo summing (Figure 1).

Assessment of echo summing and the achievable sensitivity gains using other *n* values was also performed at a fixed cycle time of 0.65 s, using NGF as a model assay (Table S4). In this experiment, a matrix blank pool consisting of an anti-NGF IA purified surrogate matrix containing NGF SIL was injected in quadruplicate for each MS condition using an injection volume of 25 μL. The correlation between experimental increases in functional S/N with echo summing and predicted S/N gain by the √*n* model is plotted in Figure 1. The square of the correlation coefficient (*R*^2^ = 0.987) is high and the slope (*y* = 1.012*x*) centered around 1.0, proving that the √*n* model can predict experimental echo-summing sensitivity gains with high correlation and minimal bias for the reference sample utilized. Furthermore, the findings suggest that reductions in dwell time from 107 ms to 21 ms, on a modern instrument, do not drastically reduce S/N calculated in the manner described. Similar findings have been documented on other MS platforms.^25^ However, shorter dwell times (<10 ms) are well-evidenced to affect the accuracy and precision of modern instruments.^26−28^

Shorter dwell times increase error in peak area due to increased peak sampling rate and collecting fewer ions, making random fluctuations in ion beam sampling more prevalent (jagged peaks). By keeping the cycle time fixed at 0.65 s and using a dwell time cutoff of ∼20 ms, a sampling rate of ∼12–18 points per 8–12 s peak and a sufficiently stable ion flux is ensured for up to ∼32 transitions collected simultaneously. Extensions of echo summing to workflows with smaller peaks and/or those needing collection of additional simultaneous transitions require establishment of similar cutoffs to preserve assay gains. Fundamental reductions in sampling rate and dwell time, outside the constraints described herein, were not interrogated but are predicted to limit the assay gains.

It is well understood that the canonical publications documenting the relationship between dwell time and S/N measured a different instrumental noise and signal prior to signal processing rather than the functional S/N measurement described here. However, as S/N measured in the way presented is the main predictor of assay LLOQ in bioanalysis, the fact that measured S/N does not decrease by the factor 1/√d*t* during these experiments leaves the door open for echo summing to provide achievable LLOQ gains.

To further characterize the improvements in LLOQ afforded by transition summing, a functional sensitivity test for the two peptides of sASGPR was performed (Figure 2 and Table 2), using a methodology similar to that published by Shuford et al.^7^ Comparisons of calibration curves and statistics for traditional versus echo summing collection of sASGPR are shown in Figures S4 and S5, as well as Tables S5 and S6. LLOQ was assessed by plotting and modeling, using an unweighted power line of best fit, the precision in QC samples, expressed as %CV in the measured sASGPR concentration across 5 replicates and interpolating the sASGPR concentration at 20% CV (Figure 2). More extensive dilution of QC samples and additional replicates (up to 256-fold, 5 replicates) was utilized in the functional sensitivity testing compared to that used in proof-of-concept NGF testing (up to 4-fold, 4 replicates) to comprehensively capture the relationship between %CV and concentration. Precision plots for each collection method are terminated when concentration readbacks reach negative values to preserve the plot trend. Tabulated QC accuracy and precision extending to 16-fold diluted serum (lowest measurable QC sample) for each collection method are presented in Table 2.

**Table 2:**
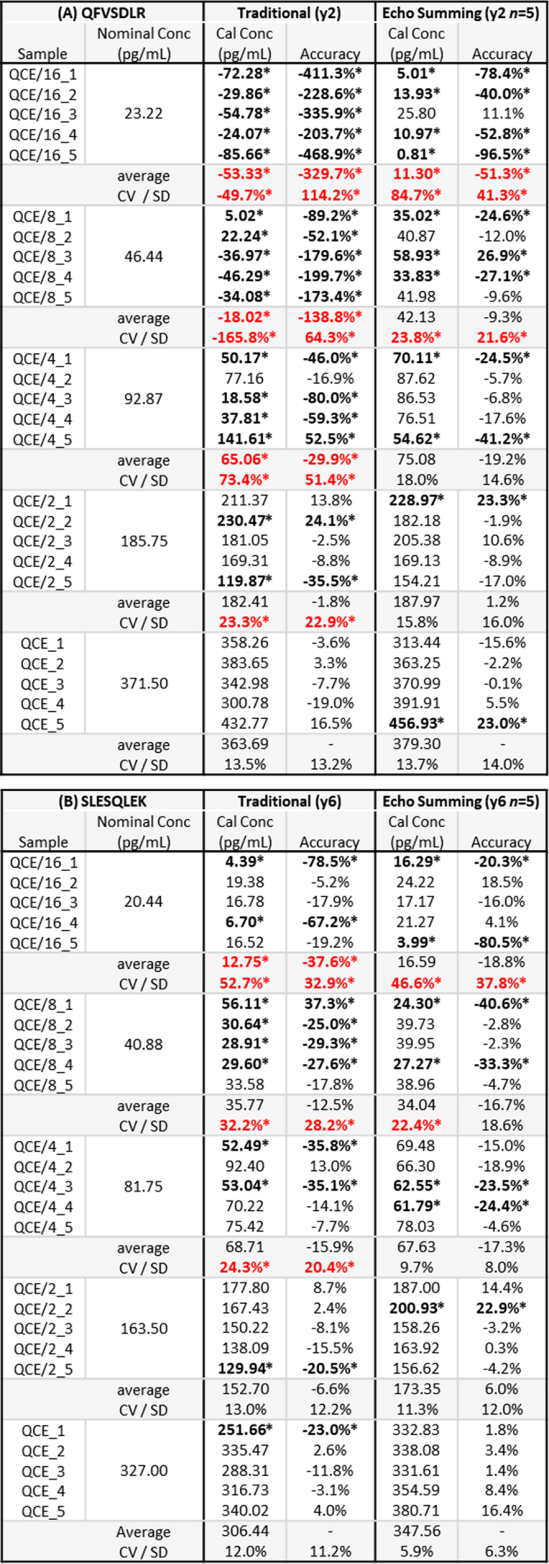
Comparison of QC Accuracy and Precision for Traditional vs Echo Summing of sASGPR Using (A) the QFVSDLR Peptide and (B) the SLESQLEK Peptide^a^

a Individual values outside of acceptance criteria are bolded and denoted by an asterisk, while average values are shown in bold red font.

The improvement in LLOQ utilizing echo summing for both peptides is 1.96-fold (247.61 vs 126.22 pg/mL; QFVSDLR) and 2.00-fold (104.83 vs 52.47 pg/mL; SLESQLEK). These LLOQ gains are consistent with the achievable improvements in S/N presented for NGF (2.08-fold) and consistent with the predicted gain of √*n* or 2.24-fold. Accuracy trends for sASGPR are also improved when echo summing more dilute serum QC samples due to the improved sensitivity (Table 2). Acceptable average accuracy and SD are preserved with echo summing at a QC concentration 4-fold lower than traditional collection for both QFVSDLR (QCE/4 vs QCE) and SLESQLEK (QCE/8 vs QCE/2). A finding that suggests echo summing improvements in assay accuracy outpace those of assay precision.

To further reinforce these trends observed when assaying diluted serum QCs, smaller volumes of starting serum (10 μL, 20 μL) were assayed without dilution in triplicate, and precision and accuracy were evaluated near the echo summing LLOQ for each peptide (Tables S7 and S8). For QFVSDLR, echo summing provides improved accuracy (−15.3 ± 32.5% vs −57.8 ± 34.9% bias) and precision (38.4% vs 82.6% CV) compared with traditional collection in the 20 μL sample (Table S7). For SLESQLEK, echo summing provides improved accuracy (−20.6 ± 16.3% vs −31.0 ± 40.1% bias) and precision (20.5% vs 58.2% CV) compared to traditional collection in the 10 μL sample (Table S8). These findings of improved low-end QC accuracy, precision, and sensitivity in both serially diluted and undiluted serum are consistent with trends previously discussed for NGF and reflect the characteristic improvements introduced through adoption of echo summing for protein quantification by sequential IA.

## Conclusion

The √*n* echo summing model was confirmed to hold true when applied to NGF and sASGPR protein quantification by sequential protein and tryptic peptide IA-nLC-MS/MS. Echo summing the top transition of NGF for five iterations led to a marked improvement compared to traditional collection in the relative average accuracy (−1.5 ± 7.7 vs −41.7 ± 10.7% bias) and precision (7.8 vs 18.4% CV) of the low-end diluted serum QC sample (*N* = 4). Echo summing the top transition of two peptides of sASGPR, QFVSDLR and SLESQLEK, for five iterations led to a proportional improvement in functional LLOQ consistent with the √*n* model of 1.96 and 2.00-fold, respectively, in serially diluted serum QC samples (*N* = 5 each population). Echo summing also extended acceptable average accuracy and SD for sASGPR to a 4-fold lower concentration in the same QC samples. Consistent improvement in accuracy and precision in undiluted serum, expressing endogenous sASGPR near the LLOQ, was also achieved with echo summing. Furthermore, collection of additional echo summing iterations returned S/N gains consistent with √*n* model for NGF suggesting that larger sensitivity increases may also be possible.

We believe that echo summing is advantageous in protein quantification where noise is reduced, and sampling rate and dwell time can generally fit the constraints described, regardless of sample preparation technique. Thus, far, no adverse effects on quantitative performance compared to traditional collection have been encountered with echo summing. Extensions of echo summing to protein quantitation lacking protein level IA for dystrophin^29^ provides additional support of this conclusion. Echo summing requires no additional cost and minimal labor to deploy, and not performing echo summing may leave considerable sensitivity gains unexploited. For these reasons, echo summing has displaced traditional collection as our default collection technique for high sensitivity quantification of proteins employing an IA enrichment step. However, the deployment of echo summing presented here will need to be assessed for concordance with other QqQ instruments.
