# Supplementary material for: Improving the Sensitivity of Protein Quantification by Immunoaffinity Liquid Chromatography—Triple Quadrupole Mass Spectrometry Using an Iterative Transition Summing Technique
Source: Anal Chem. 2024 Aug 26;96(36):14326–31. doi: 10.1021/acs.analchem.3c04598 (PMC11391403; doi:10.1021/acs.analchem.3c04598)
Supplement: Supplementary file 1 — ac3c04598_si_001.pdf [file ac3c04598_si_001.pdf]

Supporting Information

Improving the Sensitivity of Protein Quantification by Immunoaffinity Liquid Chromatography – Triple Quadrupole Mass Spectrometry Using an Iterative Transition Summing Technique

Jay S. Johnson,* Joe Palandra, Nikolaos Psychogios, Jason M, Walsh, and Hendrik Neubert
Pharmacokinetics, Dynamics & Metabolism (PDM), Pfizer, Andover, Massachusetts 01810, United States

Contents

NGF Calibration Curve and QC Sample Specifics **S2**

sASGPR Calibration Curve and QC Sample Specifics **S2**

Additional LC and MS Operating Parameters **S2**

Dwell Time Theory Predicted Sensitivity Gains Associated with Echo Summing (Figure S1) **S3**

NGF Transition Collection Parameters (Table S1) **S4**

sASGPR Transition Collection Parameters (Table S2) **S5**

NGF Calibration Curve (Figure S2) **S6**

NGF Calibration Curve Statistics (Table S3) **S7**

NGF Chromatographic Comparison (Figure S3) **S8**

Functional S/N vs *n* for NGF Echo Summing (Table S4) **S9**

sASGPR Calibration Curve (Figure S4 and S5) **S10**

sASGPR Calibration Curve Statistics (Table S5 and S6) **S12**

sASGPR Statistics for Smaller Starting Serum Volumes (Table S7 and S8) **S14**

NGF Calibration Curve and QC Sample Specifics

Calibration curves were constructed by spiking recombinant human NGF (R&D Systems: 256-GF-100/CF) into 1% milk in water (Millipore / 6250) at 0.0, 2.5, 5.0, 10.0, 20.0, 40.0, 80.0, 160.0, 320.0 pg/mL (N=2). Precision and accuracy testing was performed using QC samples prepared by serially diluting pooled human serum (BioIVT / HMSRM) or spiking with recombinant human NGF at the following 4 levels: QCE/4 (serum diluted four-fold in 1% milk), QCE/2 (serum diluted two-fold in 1% milk), QCE (serum), QCL (serum + 45 pg/mL rhNGF) (N=4). 200µL of final sample volume was transferred to an Eppendorf LoBind deep well plate for further preparation.

sASGPR Calibration Curve and QC Sample Specifics

Calibration curves were constructed by spiking recombinant human ASGPR (R&D Systems: 4394-AS) into 4% bovine serum albumin (BSA) in PBS at 0, 1.95, 3.91, 7.81, 15.63, 31.25, 62.50, 125.00, 250.00, 500.00, 1000.00, 2000.00, and 4000.00 pg/mL (N=2). Functional sensitivity assessment was performed using QC samples prepared by serially diluting pooled human male AB serum (Sigma / H4522) into 4% BSA in PBS at the following 9 levels: QCE/256 (serum diluted 256-fold in 4% BSA), QCE/128 (serum diluted 128-fold in 4% BSA), QCE/64 (serum diluted 64-fold in 4% BSA), QCE/32 (serum diluted 32-fold in 4% BSA), QCE/16 (serum diluted 16-fold in 4% BSA), QCE/8 (serum diluted 8-fold in 4% BSA), QCE/4 (serum diluted 4-fold in 4% BSA), QCE/2 (serum diluted 2-fold in 4% BSA), QCE (serum) (N=5). 80µL of final sample volume was transferred to an Eppendorf LoBind deep well plate for further preparation. An additional parallelism check consisting of 10 and 20µL of human serum (N=3) without additional surrogate matrix was also transferred for further preparation.

Additional LC and MS Operating Parameters

For analysis of NGF, 85µL of sample volume resulting from protein IA (R&D / AF256, biotinylated in-house) followed by alkylation, reduction, and tryptic digestion in the presence of 0.9 fmol/µL of stable isotope labelled (SIL) IDTACVCVLSR^ (^ = $^{13}C_6$, $^{15}N_4$) was injected onto the LC system described in the publication operating in an online peptide IA configuration[12-14].

For analysis of sASGPR, 40µL of sample volume resulting from protein IA (R&D / MAB4394, biotinylated in-house) followed by alkylation, reduction, and tryptic digestion in the presence of 7.2 fmol/ µL of SIL SLESQLEK^ (^ = $^{13}C_6$, $^{15}N_2$ and QFVSDLR^ (^ = $^{13}C_6$, $^{15}N_4$) was injected onto the LC system described in the publication operating in an online peptide IA configuration[12-14].

The nLC column utilized in all analysis was an EASY-Spray variant (PepMap C18, 15cm x 75µm, 3µm, 100Å, Thermo Fisher Scientific) operating at 600nL/min and 65˚C.

The eluate from the nLC column was introduced into a TSQ Altis QqQ (Thermo Fisher Scientific; Waltham, MA) outfitted with an EASY-Spray nLC ESI source operating under the following source conditions for all analysis: capillary voltage +3000V, ion transfer tube temperature 300˚C, CID Gas 2 mTorr, Source Fragmentation 0V, Chromatographic Peak Width 20 sec, chromatographic filter on. For analysis of NGF, QqQ resolution was set to Q1 resolution (FWHM) 0.7 Da, Q3 resolution (FWHM) 0.4 Da. For analysis of sASGPR, QqQ resolution was set to Q1 resolution (FWHM) 0.4 Da, Q3 resolution (FWHM) 0.2 Da.

Dwell Time Theory Predicted Sensitivity Gains Associated with Echo Summing

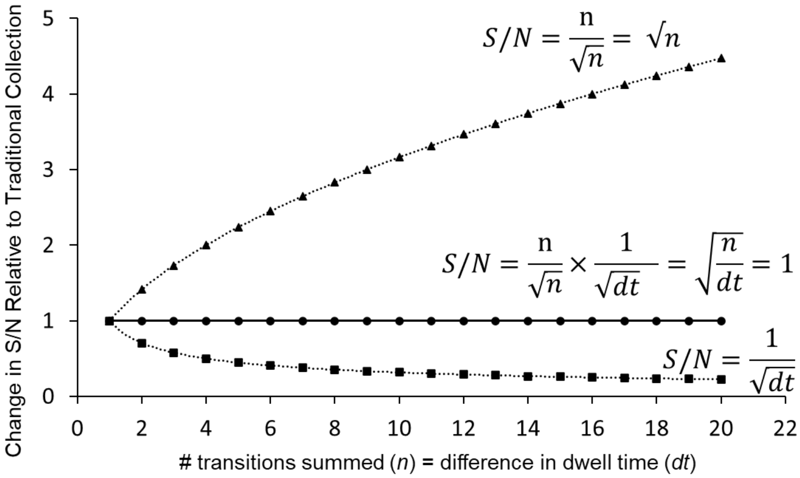

Figure S1. Increases in S/N due to echo summing *n* iterations are predicted by dwell time theory to be offset by the decrease in S/N resulting from the faster scan speed or *dt*

NGF Transition Collection Parameters

Traditional Transition Collection							
#	Transition Name	Usage	Precursor (m/z)	Product (m/z)	Collision Energy (V)	Cycle Time (sec)	Dwell Time (msec)
1	IDTACVCVLSR y6	qualitative	647.318	733.403	22.5	0.65	107
2	IDTACVCVLSR y7	quantitative		893.433			
3	IDTACVCVLSR y8	qualitative		964.470			
4	IDTACVCVLSR^ y6 (SIL)	normalization	653.834	746.433			
5	IDTACVCVLSR^ y7 (SIL)	normalization		906.464			
6	IDTACVCVLSR^ y8 (SIL)	normalization		977.501			
**Echo Summing Transition Collection (*n = 5, dt = 2.36*)**							
#	Transition Name	Usage	Precursor (m/z)	Product (m/z)	Collision Energy (V)	Cycle Time (sec)	Dwell Time (msec)
1	IDTACVCVLSR y6	qualitative	647.318	733.403	22.5	0.65	45
2	IDTACVCVLSR y7 -b	quantitative		893.425			
3	IDTACVCVLSR y7 -a	quantitative		893.429			
4	IDTACVCVLSR y7	quantitative		893.433			
5	IDTACVCVLSR y7 +a	quantitative		893.437			
6	IDTACVCVLSR y7 +b	quantitative		893.441			
7	IDTACVCVLSR y8	qualitative		964.470			
8	IDTACVCVLSR^ y6 (SIL)	normalization	653.834	746.433			
9	IDTACVCVLSR^ y7 -b (SIL)	normalization		906.456			
10	IDTACVCVLSR^ y7 -a (SIL)	normalization		906.460			
11	IDTACVCVLSR^ y7 (SIL)	normalization		906.464			
12	IDTACVCVLSR^ y7 +a (SIL)	normalization		906.468			
13	IDTACVCVLSR^ y7 +b (SIL)	normalization		906.472			
14	IDTACVCVLSR^ y8 (SIL)	normalization		977.501			

Table S1. Comparison of traditional (top) versus echo summing (bottom) transition collection tables for the quantification of NGF.

sASGPR Transition Collection Parameters

#	Transition Name	Usage	Precursor (m/z)	Product (m/z)	Collision Energy (V)	Cycle Time (sec)	Dwell Time (msec)
**Traditional Transition Collection**							
1	QFVSDLR y2	quantitative		288.203	17.8		
2	QFVSDLR y4	qualitative	432.732	490.262	14.8		
3	QFVSDLR y5	qualitative		589.330	14.8		
4	QFVSDLR^ y2 (SIL)	normalization		298.211	17.8		
5	QFVSDLR^ y4 (SIL)	normalization	437.736	500.270	14.8		
6	QFVSDLR^ y5 (SIL)	normalization		599.339	14.8		
7	SLESQLEK y3	qualitative		389.239	19.7	0.65	53
8	SLESQLEK y5	qualitative	467.248	604.330	14.7		
9	SLESQLEK y6	quantitative		733.373	14.7		
10	SLESQLEK^ y3 (SIL)	normalization		397.254	19.7		
11	SLESQLEK^ y5 (SIL)	normalization	471.255	612.344	14.7		
12	SLESQLEK^ y6 (SIL)	normalization		741.387	14.7		
**Echo Summing Transition Collection (*n = 5, dt = 2.38*)**							
**#**	**Transition Name**	**Usage**	**Precursor (m/z)**	**Product (m/z)**	**Collision Energy (V)**	**Cycle Time (sec)**	**Dwell Time (msec)**
1	QFVSDLR y2 -b	quantitative		288.195	17.8		
2	QFVSDLR y2 -a	quantitative		288.199	17.8		
3	QFVSDLR y2	quantitative		288.203	17.8		
4	QFVSDLR y2 +a	quantitative	432.732	288.207	17.8		
5	QFVSDLR y2 +b	quantitative		288.211	17.8		
6	QFVSDLR y4	qualitative		490.262	14.8		
7	QFVSDLR y5	qualitative		589.330	14.8		
8	QFVSDLR^ y2 -b (SIL)	normalization		298.203	17.8		
9	QFVSDLR^ y2 -a (SIL)	normalization		298.207	17.8		
10	QFVSDLR^ y2 (SIL)	normalization		298.211	17.8		
11	QFVSDLR^ y2 +a (SIL)	normalization	437.736	298.215	17.8		
12	QFVSDLR^ y2 +b (SIL)	normalization		298.219	17.8		
13	QFVSDLR^ y4 (SIL)	normalization		500.270	14.8		
14	QFVSDLR^ y5 (SIL)	normalization		599.339	14.8	0.65	22
15	SLESQLEK y3	qualitative		389.239	19.7		
16	SLESQLEK y5	qualitative		604.330	14.7		
17	SLESQLEK y6 -b	quantitative		733.365	14.7		
18	SLESQLEK y6 -a	quantitative	467.248	733.369	14.7		
19	SLESQLEK y6	quantitative		733.373	14.7		
20	SLESQLEK y6 +a	quantitative		733.377	14.7		
21	SLESQLEK y6 +b	quantitative		733.381	14.7		
22	SLESQLEK^ y3 (SIL)	normalization		397.254	19.7		
23	SLESQLEK^ y5 (SIL)	normalization		612.344	14.7		
24	SLESQLEK^ y6 –b (SIL)	normalization		741.379	14.7		
25	SLESQLEK^ y6 –a (SIL)	normalization	471.255	741.383	14.7		
26	SLESQLEK^ y6 (SIL)	normalization		741.387	14.7		
27	SLESQLEK^ y6 +a (SIL	normalization		741.391	14.7		
28	SLESQLEK^ y6 +b (SIL)	normalization		741.395	14.7		

Table S2. Comparison of traditional (top) versus echo summing (bottom) transition collection tables for the quantification of sASGPR.

NGF Calibration Curve

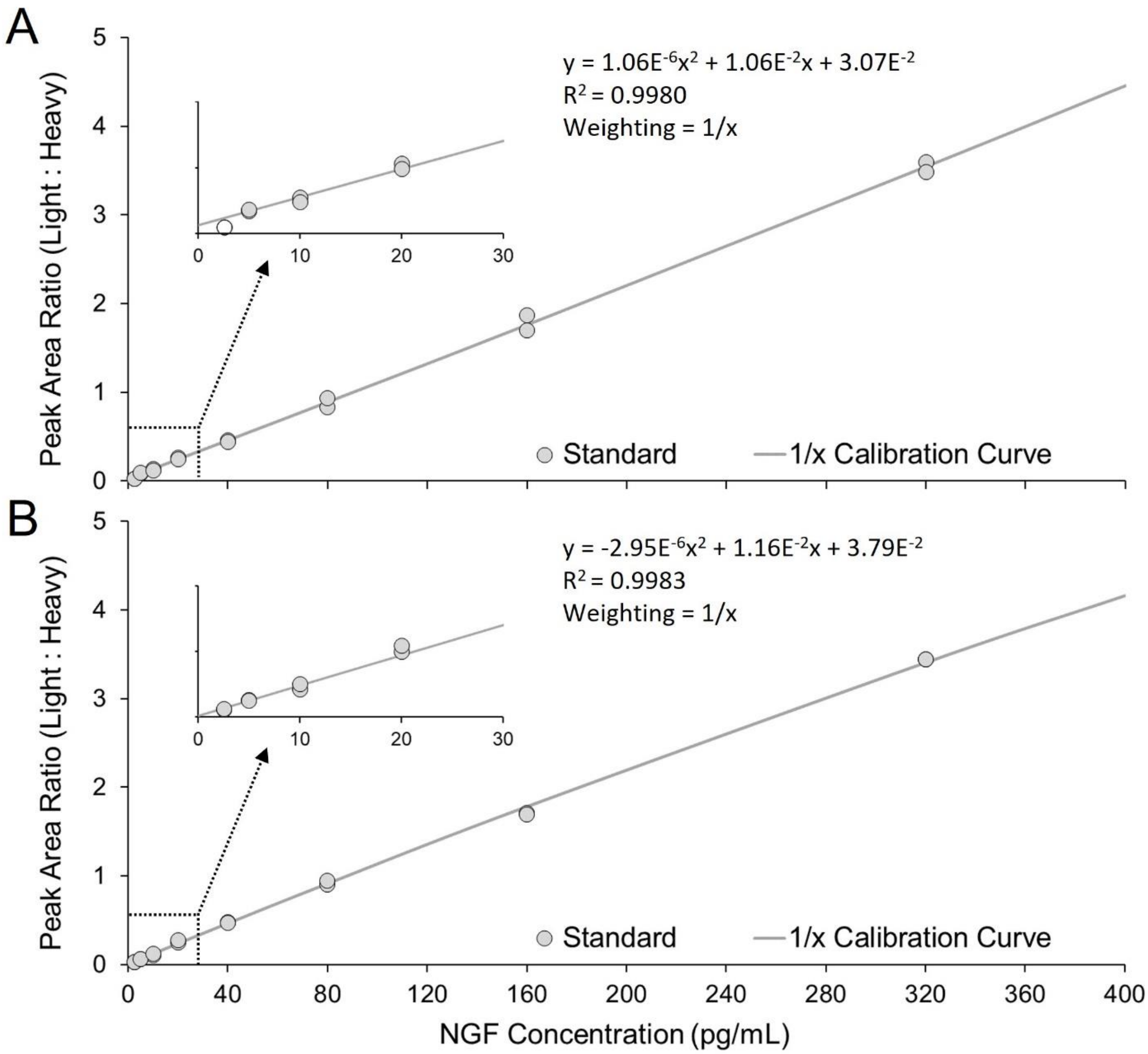

Figure S2. Comparison of the calibration curve plots for traditional (A) vs echo summing (B) transition collection for the quantification of NGF. Calibrators that fail to meet the accuracy acceptance criteria of ± 20% are excluded from the calibration curve and denoted by open circles in the graph. The lower calibration range is determined by the lowest calibrator for which both replicates meet the accuracy acceptance criteria.

NGF Calibration Curve Statistics

NGF		Traditional (y7)		Echo Summing (y7 *n*=5)	
Sample	Actual Conc (pg/mL)	Cal Conc (pg/mL)	Accuracy	Cal Conc (pg/mL)	Accuracy
CAL1_1	0.00	**-2.17***	-	**0.05***	-
CAL2_1	2.50	**-0.63***	**-125.2%***	2.02	-19.2%
CAL3_1	5.00	5.04	0.8%	5.39	7.9%
CAL4_1	10.00	9.91	-0.9%	8.87	-11.3%
CAL5_1	20.00	21.93	9.6%	21.17	5.8%
CAL6_1	40.00	39.89	-0.3%	41.35	3.4%
CAL7_1	80.00	74.96	-6.3%	79.03	-1.2%
CAL8_1	160.00	169.51	5.9%	153.72	-3.9%
CAL9_1	320.00	324.03	1.3%	323.52	1.1%
CAL1_2	0.00	**-2.46***	-	**-0.20***	-
CAL2_2	2.50	**-0.62***	**-124.8%***	2.38	-4.8%
CAL3_2	5.00	5.64	12.9%	5.06	1.1%
CAL4_2	10.00	8.21	-17.9%	10.47	4.7%
CAL5_2	20.00	19.96	-0.2%	23.23	16.2%
CAL6_2	40.00	38.21	-4.5%	40.75	1.9%
CAL7_2	80.00	83.95	4.9%	83.32	4.1%
CAL8_2	160.00	154.13	-3.7%	151.77	-5.1%
CAL9_2	320.00	314.61	-1.7%	323.23	1.0%

Table S3. Comparison of the calibration curve statistics for traditional vs echo summing transition collection for NGF. Values outside of acceptance criteria are bolded red and denoted by an asterisk.

NGF Chromatographic Comparison

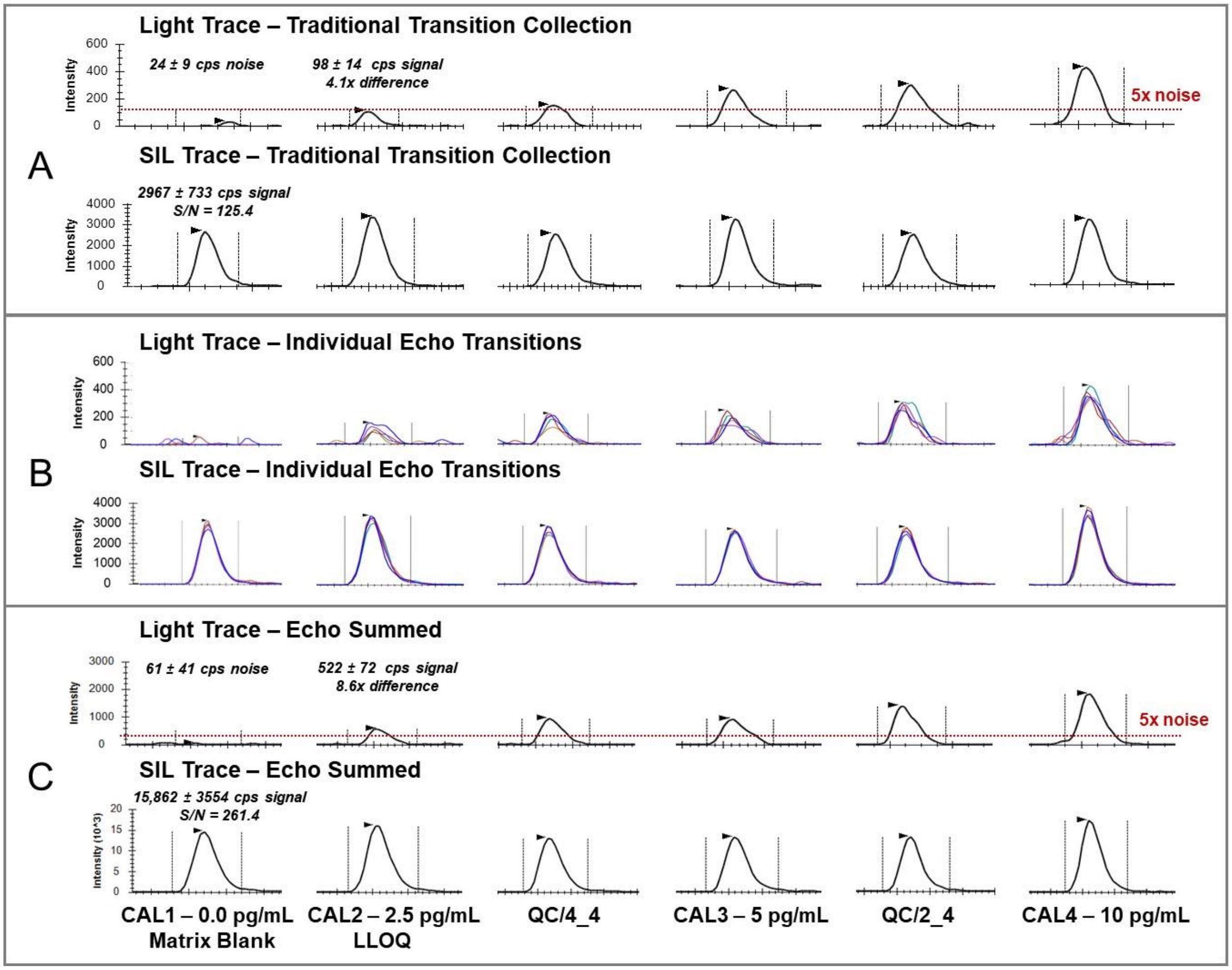

Figure S3. Extracted chromatograms of the light and SIL y7 transition of NGF collected traditionally as a single transition (A) collected as five individual echo transitions (B) and the echo summing of the five individual echo transitions from panel B (C). Signal counts presented are mean values generated from replicate runs (N=5 for matrix blank, N=2 for CAL2). Chromatograms chosen for depiction are those which have the highest correlation to the mean values.

Functional S/N vs *n* for NGF Echo Summing

Cycle Time (sec)	# transitions	Dwell Time (msec)	*n*	dt	Avg Signal Height (cps)	Signal Gain	Avg Signal Noise (cps)	Avg S/N	Increase S/N	Model Increase S/N
0.65	6	107	1	1.00	531	1.0	24	22	1.00	1.00
0.65	10	64	3	1.68	1469	2.8	34	43	1.95	1.73
0.65	14	45	5	2.36	2229	4.2	41	55	2.47	2.24
0.65	18	35	7	3.06	2570	4.8	41	63	2.85	2.65
0.65	22	29	9	3.74	2999	5.6	58	52	2.35	3.00
0.65	26	24	11	4.44	3422	6.4	47	73	3.28	3.32
0.65	30	21	13	5.15	3414	6.4	39	87	3.94	3.61

Table S4. Experimental parameters utilized and data generated when assessing echo summing performance of the y7 transition of NGF with additional *n* values in a fixed cycle time of 0.65 seconds.

sASGPR Calibration Curve

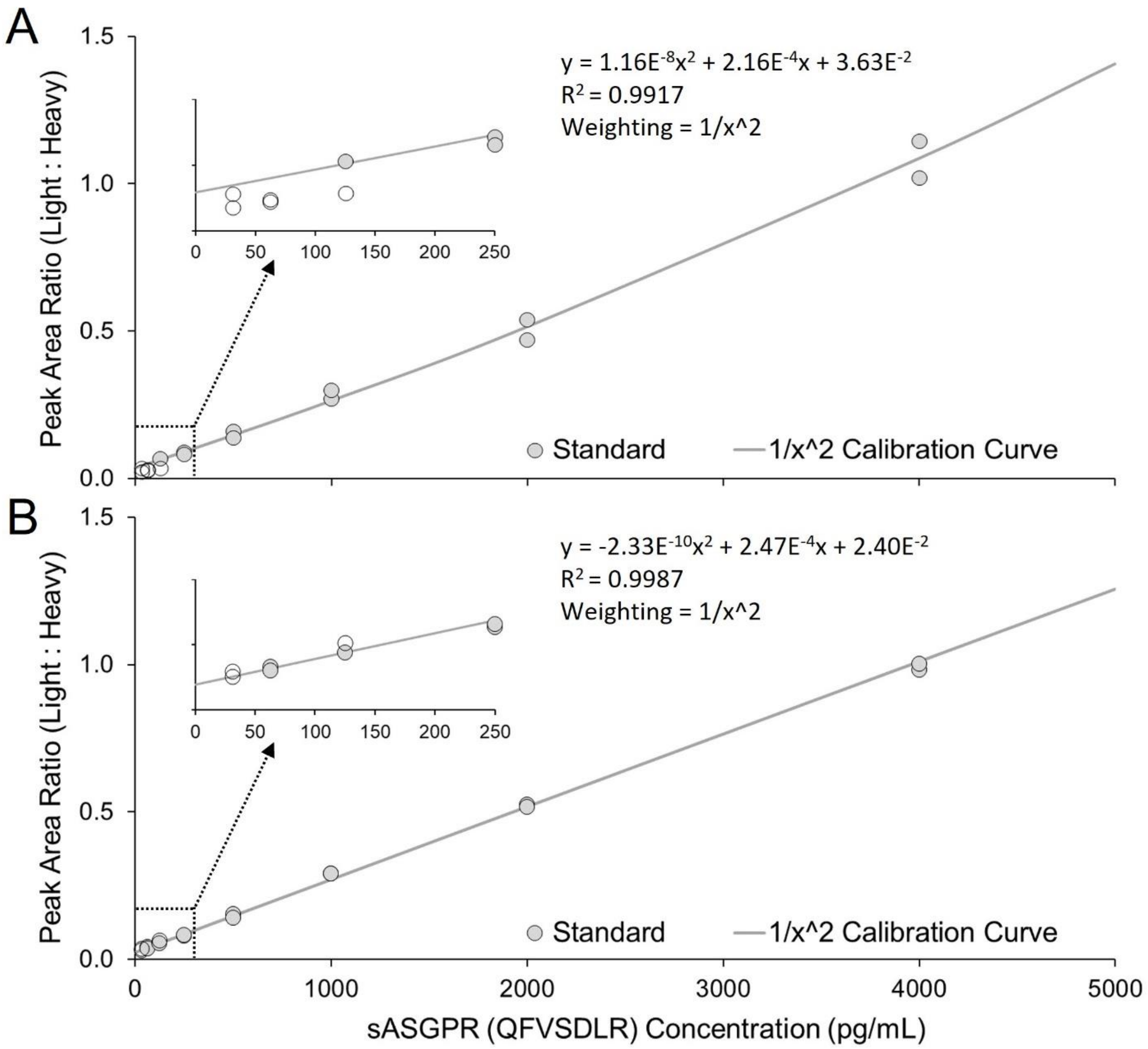

Figure S4. Comparison of the calibration curve plots for traditional (A) vs echo summing (B) transition collection for the quantification of sASGPR using QFVSDLR peptide. Calibrators that fail to meet the accuracy acceptance criteria of ± 20% are excluded from the calibration curve and denoted by open circles in the graph. The lower calibration range is determined by the lowest calibrator for which both replicates meet the accuracy acceptance criteria.

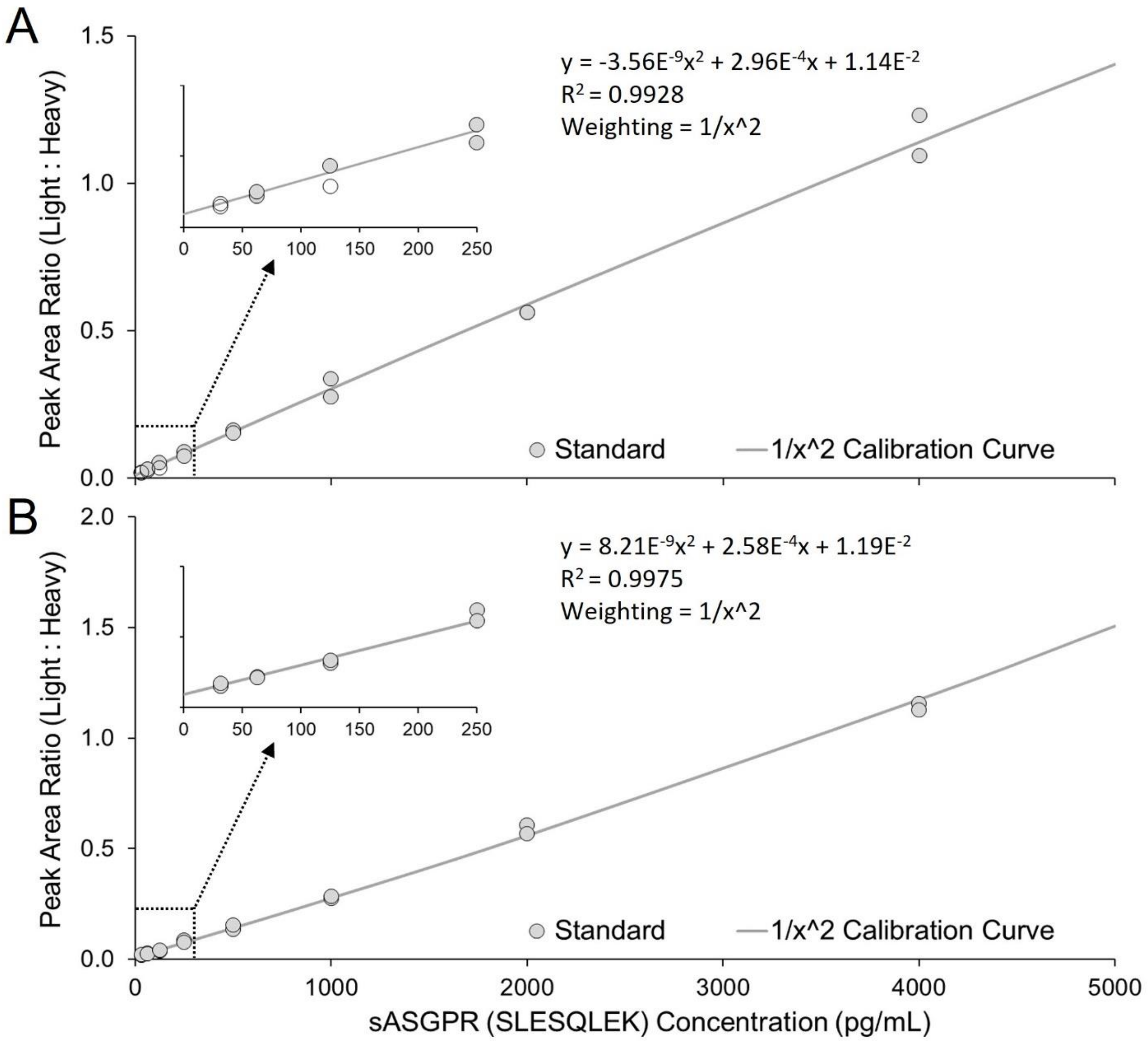

Figure S5. Comparison of the calibration curve plots for traditional (A) vs echo summing (B) transition collection for the quantification of sASGPR using SLESQLEK peptide. Calibrators that fail to meet the accuracy acceptance criteria of ± 20% are excluded from the calibration curve and denoted by open circles in the graph. The lower calibration range is determined by the lowest calibrator for which both replicates meet the accuracy acceptance criteria.

sASGPR Calibration Curve Statistics

QFVSDLR		Traditional (y2)		Echo Summing (y2 *n*=5)	
Sample	Actual Conc (pg/mL)	Cal Conc (pg/mL)	Accuracy	Cal Conc (pg/mL)	Accuracy
CAL0_1	0.00	**-55.16***	-	**-8.74***	-
CAL5_1	31.25	**-65.20***	**-308.6%***	**33.45**	**7.0%**
CAL6_1	62.50	**-40.02***	**-164.0%***	70.99	13.6%
CAL7_1	125.00	135.55	8.4%	**163.23***	**30.6%***
CAL8_1	250.00	240.43	-3.8%	223.60	-10.6%
CAL9_1	500.00	551.83	10.4%	527.05	5.4%
CAL10_1	1000.00	1019.10	1.9%	1079.90	8.0%
CAL11_1	2000.00	1825.70	-8.7%	2025.80	1.3%
CAL12_1	4000.00	4186.70	4.7%	3886.20	-2.8%
CAL0_2	0.00	**-49.10***	-	**-0.82***	-
CAL5_2	31.25	**-4.34***	**-113.9%***	**51.77**	**65.7%***
CAL6_2	62.50	**-31.52***	**-150.4%***	55.83	-10.7%
CAL7_2	125.00	**-0.56***	**-100.4%***	125.79	0.6%
CAL8_2	250.00	207.18	-17.1%	234.69	-6.1%
CAL9_2	500.00	453.68	-9.3%	472.35	-5.5%
CAL10_2	1000.00	1141.50	14.2%	1076.60	7.7%
CAL11_2	2000.00	2085.90	4.3%	1996.10	-0.2%
CAL12_2	4000.00	3784.70	-5.4%	3974.40	-0.6%

Table S5. Comparison of the calibration curve statistics for traditional vs echo summing transition collection for the quantification of sASGPR using QFVSDLR peptide. Values outside of acceptance criteria are bolded red and denoted by an asterisk. Calibrators below 31.25 pg/mL could not be quantified and are excluded from data reporting.

SLESQLEK		Traditional (y6)		Echo Summing (y6 *n*=5)	
Sample	Actual Conc (pg/mL)	Cal Conc (pg/mL)	Accuracy	Cal Conc (pg/mL)	Accuracy
CAL0_1	0.00	**-2.60***	-	**18.08***	-
CAL5_1	31.25	**23.86***	**-23.6%***	28.34	-9.3%
CAL6_1	62.50	54.26	-13.2%	60.28	-3.6%
CAL7_1	125.00	144.24	15.4%	106.92	-14.5%
CAL8_1	250.00	267.85	7.1%	286.79	14.7%
CAL9_1	500.00	518.46	3.7%	473.20	-5.4%
CAL10_1	1000.00	907.41	-9.3%	994.03	-0.6%
CAL11_1	2000.00	1905.90	-4.7%	2163.00	8.1%
CAL12_1	4000.00	4343.60	8.6%	3940.00	-1.5%
CAL0_2	0.00	**-23.53***	-	**-11.42***	-
CAL5_2	31.25	**33.55**	**7.4%**	37.20	19.1%
CAL6_2	62.50	67.12	7.4%	56.59	-9.5%
CAL7_2	125.00	**84.47***	**-32.4%***	115.67	-7.5%
CAL8_2	250.00	213.02	-14.8%	249.59	-0.2%
CAL9_2	500.00	485.33	-2.9%	546.53	9.3%
CAL10_2	1000.00	1117.40	11.7%	1027.40	2.7%
CAL11_2	2000.00	1906.30	-4.7%	2028.00	1.4%
CAL12_2	4000.00	3829.60	-4.3%	3849.10	-3.8%

Table S6. Comparison of the calibration curve statistics for traditional vs echo summing transition collection for the quantification of sASGPR using SLESQLEK peptide. Values outside of acceptance criteria are bolded red and denoted by an asterisk. Calibrators below 31.25 pg/mL could not be quantified and are excluded from data reporting.

sASGPR Statistics for Smaller Starting Serum Volumes

QFVSDLR		Traditional (y2)		Echo Summing (y2 *n*=5)	
Sample	Nominal Conc (pg/mL)	Cal Conc (pg/mL)	Accuracy	Cal Conc (pg/mL)	Accuracy
QCE_10uL_1	46.44	**-22.72***	**-148.9%***	**27.00***	**-41.9%***
QCE_10uL_2		**-14.48***	**-131.2%***	47.06	1.3%
QCE_10uL_3		**-11.52***	**-124.8%***	49.88	7.4%
	average	**-16.24***	**-135.0%***	41.31	-11.0%
	CV / SD	**-35.7%***	12.5%	**30.2%***	**26.9%***
QCE_20uL_1	92.87	**28.85***	**-68.9%***	89.74	-3.4%
QCE_20uL_2		75.53	-18.7%	101.74	9.5%
QCE_20uL_3		**13.29***	**-85.7%***	**44.48**	**-52.1%***
	average	**39.22***	**-57.8%***	78.65	-15.3%
	CV / SD	**82.6%***	**34.9%***	**38.4%***	**32.5%***

Table S7. Comparison of the precision (expressed as the CV in the measured sASGPR concentration using QFVSDLR peptide) and accuracy (expressed as the mean bias ± SD from expected sASGPR concentration using QFVSDLR peptide) across 3 replicates of 10 or 20uL of undiluted serum collected by traditional vs echo summing transition collection. Individual values outside of acceptance criteria are bolded and denoted by an asterisk while average values are bolded red.

SLESQLEK		Traditional (y6)		Echo Summing (y6 *n*=5)	
Sample	Nominal Conc (pg/mL)	Cal Conc (pg/mL)	Accuracy	Cal Conc (pg/mL)	Accuracy
QCE_10uL_1	40.88	**15.91***	**-61.1%***	**25.81***	**-36.9%***
QCE_10uL_2		**21.89***	**-46.5%***	**32.37***	**-20.8%***
QCE_10uL_3		46.84	14.6%	39.12	-4.3%
	average	**28.21***	**-31.0%***	**32.44***	**-20.6%***
	CV / SD	**58.2%***	**40.1%***	**20.5%***	16.3%
QCE_20uL_1	81.75	**55.80***	**-31.7%***	72.31	-11.6%
QCE_20uL_2		91.36	11.8%	83.52	2.2%
QCE_20uL_3		**57.24***	**-30.0%***	**54.77***	**-33.0%***
	average	68.14	-16.7%	70.20	-14.1%
	CV / SD	**29.5%***	**24.6%***	**20.6%***	17.7%

Table S8. Comparison of the precision (expressed as the CV in the measured sASGPR concentration using SLESQLEK peptide) and accuracy (expressed as the mean bias ± SD from expected sASGPR concentration using SLESQLEK peptide) across 3 replicates of 10 or 20uL of undiluted serum collected by traditional vs echo summing transition collection. Individual values outside of acceptance criteria are bolded and denoted by an asterisk while average values are bolded red.
